# The United States and Canada as a coupled epidemiological system: An example from hepatitis A

**DOI:** 10.1186/1471-2334-8-23

**Published:** 2008-02-28

**Authors:** Raluca Amariei, Allan R Willms, Chris T Bauch

**Affiliations:** 1 Department of Mathematics and Statistics, The University of Guelph, Guelph, Canada

## Abstract

**Background:**

Hepatitis A (HA) is a low-incidence, non-endemic disease in Canada and the United States (US). However, a large difference in HA incidence between Canada and HA-endemic countries has made travel an important contributor to hepatitis A prevalence in Canada. There is also a (smaller) incidence differential between Canada and the US. Although the US has only moderately higher HA incidence, the volume of travel by Canadians to the US is many times higher than travel volume to endemic countries. Hence, travel to the US may constitute a source of low to moderate risk for Canadian travelers. To our knowledge, travel to the US has never been included as a potential risk factor for HA infection in Canadian epidemiologic analyses. The objective of this study was to use dynamic models to investigate the possible effects on hepatitis A incidence in Canada due to (1) implementing vaccination in the US, and (2) varying the volume of travel by Canadians to the US.

**Methods:**

We developed and analyzed age-structured compartmental models for the transmission and vaccination of hepatitis A, for both Canada and the US. Models were parameterized using data on seroprevalence, case reporting, and travel patterns. The potential effect of hepatitis A prevalence in the US on hepatitis A prevalence in Canada was captured through a term representing infection of Canadians due to travel in the US.

**Results:**

The model suggests that approximately 22% of HA cases in Canada in the mid 1990s may have been attributable to travel to the US. A universal vaccination programme that attained 70% coverage in young children in the US in the mid 1990s could have reduced Canadian incidence by 21% within 5 years.

**Conclusion:**

Since not all necessary data were available to parameterize the model, the results should be considered exploratory. However, the analysis shows that, under plausible assumptions, the US may be more important for determining HA prevalence in Canada than is currently supposed. As international travel continues to grow, making vaccination policies ever more relevant to populations beyond a country's borders, such multi-country models will most likely come into wider use as predictive aids for policy development.

## Background

Wealth varies dramatically across countries, and with it, the disease burden for many infectious diseases [1]. One example is HIV, where prevalence is 6% in sub-Saharan Africa but only 0.3% in Western Europe [2]. A less striking but still significant example is hepatitis A (HA). HA is a non-endemic, low-incidence disease in the US and Canada, but is highly endemic in many other countries [3-6]. The average reported HA incidence in Canada was 6.3 per 100,000 per year from 1980 to 1994 [7], and the average reported HA incidence in the US was 10.5 per 100,000 per year from 1980 to 1999 [8]. By comparison, in 1990, reported incidence ranged from 20 to 60 per 100,000 per year in Africa and the Middle East (depending on the country), 10 to 30 in Asia, and 20 to 40 in Central and South America [9]. Moreover, reported incidence significantly underestimates actual incidence due to under-reporting and subclinical infection [6,8,10]. Because subclinical infection is more common in children, who are infected more frequently in developing countries than developed countries, the global differential in true infection levels is much higher than for reported incidence.

This incidence differential between Canada and HA-endemic countries, combined with increasing air travel, makes travel by Canadian residents to HA-endemic countries a significant source of HA infection in Canada [11,12]. Travel to endemic countries is also a source of infection in the US, with 10% of reported infections attributable to travel in HA-endemic countries in 2001 [13].

Hepatitis A vaccine has been available in Canada and the US since 1995 [14]. In Canada, the average reported incidence from 1995 to 2003, while a targeted vaccination programme was in place, declined to 3.8 per 100,000 per year [7]. The vaccination policy in Canada is still targeted and includes high-risk groups, such as men who have sex with men, intravenous drug users, members of First Nations communities, and travelers to endemic countries, among others. In the US, after vaccination was implemented (with universal vaccination in the states with highest incidence), the reported incidence had declined to 3.7 per 100,000 per year by 2001 [14,15]. The true incidence of infection (including both clinical and subclinical infection) has been underestimated by approximately 8-fold in Canada and 10-fold in the US [8,16].

**Figure 1:**
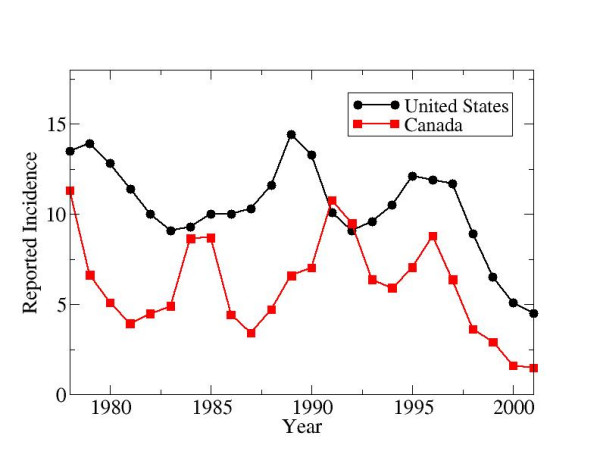
Reported incidence (per 100,000 per year) of hepatitis A infection in the United States and Canada, 1978–2001.

There also exists an incidence differential between Canada and the US, with the US having somewhat higher incidence (Figure 1). Hepatitis A incidence tends to rise and fall at the same time in the US and Canada. In fact, the reported incidence in the two countries is positively correlated with a correlation coefficient +0.54 (Figure 1) [7,8]. Interestingly, outbreaks of hepatitis A in men who have sex with men (MSM) in Montreal often follow outbreaks in MSM in New York by 1 or 2 months (Vladimir Gilca, Institut national de santé publique du Québec, pers. comm.). The two countries are also bound together by very high travel volume. For instance, in 1995, the number of person-trips by Canadian residents returning to Canada from the US was 4 times the number to all other countries combined, and 9 times the number made to all HA-endemic countries combined [16]. Similarly, on average from 1987–2006, outbound travel from Canada to the US was 10 times that to all other countries combined [17].

Given these observations, it is worth posing the question: how does HA epidemiology in the US influence HA epidemiology in Canada? This issue has implications for policy, since it implies that health interventions in one country may potentially influence health outcomes in other countries. This question also has implications not only for hepatitis A but for many diseases such as SARS, as burgeoning air travel turns local problems into global problems. In this paper, we develop mathematical (age-structured compartmental) models of hepatitis A transmission and vaccination in Canada and the US. We use travel data to couple the two countries epidemiologically through travel. We focus on these two countries (rather than attempting a global model) because of the relatively good availability of data for the US and Canada and the close relationship of Canada to the US. The coupled model allows us to analyze how transmission and vaccination in the US may be affecting HA incidence in Canada.

## Methods

We begin with a description of hepatitis A epidemiology, which will motivate our choice of mathematical model. In Canada and the US, unlike most developing countries, hepatitis A is transmitted mostly by person-to-person contact, by the fecal-oral route [10]. Unlike in many countries, foodborne outbreaks are very infrequent in Canada [18]. Children play an important role in transmission due to their higher rates of subclinical infection and poor hygiene [19]. Clinical illness typically lasts four weeks, there is no chronic state of infection [20], and natural immunity is lifelong. Although 70% of infected post-adolescents develop jaundice, many do not seek medical attention [10,21]. Individuals with symptomatic HA infection experience nausea, loss of appetite, fatigue, fever, abdominal pain and jaundice [21]. Hepatitis A infection is more severe in older individuals or those with co-morbidities such as chronic liver disease [22,23]. The most serious possible complication of hepatitis A infection is fulminant hepatic failure. The rate of mortality attributable to HA varies from 0.2% in symptomatic young adults to 1.7% in symptomatic individuals 60 years and older [24].

Given the predominance of person-to-person contact, lifelong immunity, and the importance of children in transmission, a suitable mathematical model is an age-structured compartmental model. This widely-used class of models has been shown to be particularly useful in assessing the effects of universal vaccination programmes against diseases with acquired immunity transmitted horizontally through person-to-person contact, and has been shown to provide good agreement with pre- and post-vaccination age stratified case reports and seroprevalence surveys for infectious diseases such as measles [25,26].

**Figure 2:**
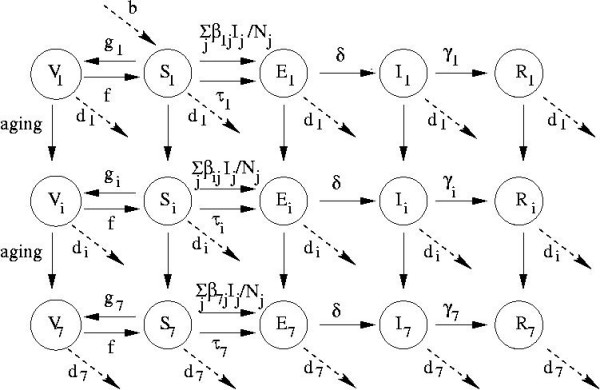
Schematic diagram of model with flows between compartments; see text for definitions of variables and parameters.

Our age-structured SEIRV compartmental model stratifies individuals according to epidemiologic status (Susceptible-Exposed-Infectious-Recovered-Vaccinated) and age class (ages 0–4, 5–9, 10–19, 20–29, 30–39, 40–59, 60+). Age classes are chosen to reflect age categories in available sources of demographic and epidemiologic data. Flow rates between compartments are defined by model parameters. Exposed individuals enter the infectious compartment at rate *δ*, and infectious individuals of age class *i *enter the recovered compartment at rate *γ*_*i*_, thereafter retaining lifelong immunity. Individuals in age class *i *are vaccinated at per capita rate $g_{i}^{US}$ thereby entering the vaccinated compartment. The 'US' superscript denotes that this parameter value is specific to the US. Vaccinated individuals lose their immunity at per capita rate *f*, re-entering the susceptible compartment. Individuals are born susceptible at rate *b*^*US*^, die at rate $d_{i}^{US}$, and age at rate *a*_i_. Maternal immunity is short-lived and affects relatively few individuals in a non-endemic country such as Canada, so we do not include it [10]. Since HA in Canada and the US is spread primarily person-to-person, we do not model foodborne or waterborne outbreaks [10]. The model equations appear in Appendix A and the parameterization is described in Appendix B. A diagram of the model appears in Figure 2.

The United States and Canada are both large countries and one may consider that a model by states would be more appropriate. However, despite their close proximity, there is more travel within Canada than between Canada and the US: from 1998 to 2004, there were 4.6 times as many person-trips made within Canada (across provinces) as between Canada and the US [17].

Table 1 gives the parameter values used for the US model and their data sources [8,13,24,27-30]. Demographic and epidemiologic parameter values are from the pre-vaccine era, 1980–1994. Demographic parameters such as birth rates and age-specific death rates were taken from demographic data. Clinical and epidemiologic literature on hepatitis A were used to determine the durations of latent and infectious periods, vaccine efficacy, and duration of vaccine-derived immunity. The rate at which a susceptible person is infected due to travel in endemic countries ($\tau_{i}^{US}$), and the rate at which a susceptible person is infected by infectious persons due to domestic US transmission ($\beta_{ij}^{US}$) were computed simultaneously using: (1) published data on the true incidence of hepatitis A in the US, adjusted for under-reporting and the probability of jaundice [8], (2) data on the age-specific proportion of cases attributable to travel in endemic countries [13], and (3) an assumed form for a "Who Acquires Infection From Whom" matrix consisting of the $\beta_{ij}^{US}$ parameters [31]. This method of computation, which uses the model equations and does not require explicit knowledge of the force of infection or seroprevalence data (although those were necessary to estimate the true incidence in this particular case), is described in Appendix B.

**Table 1:** Definitions of parameters and variables, and parameter values used for the age-structured US model.*

Variable/Parameter	Definition	Values	Reference
$S_{i}^{US}$	Number of susceptible individuals in the United States at a given time *t *in age class *i*	N/A (not applicable)	N/A
$E_{i}^{US}$	Number of exposed (infected but not yet infectious) individuals in the United States at a given time *t *in age class *i*	N/A	N/A
$I_{i}^{US}$	Number of infectious individuals in the United States at a given time *t *in age class *i*	N/A	N/A
$R_{i}^{US}$	Number of recovered individuals in the United States at a given time *t *in age class *i*	N/A	N/A
$V_{i}^{US}$	Number of vaccinated individuals in the United States at a given time *t *in age class *i*	N/A	N/A
*γ *_*i*_	Mean duration of infectious period	3.5, 3.0, 2.5, 2.5, 2.5, 2.5, 2.5 weeks	[27,28]
*δ*	Mean duration of latent period	2 weeks	[27,29]
*f*	Rate of waning vaccine-derived immunity	0.58% per year	[24]
*a*_i_	Rate at which an individual in age class *i *ages (enters age class *i*+1)	0.2, 0.2, 0.1, 0.1, 0.1, 0.05, 0 per year for age classes 1, 2, ... 7	N/A
*b*^US^	Birth rate	0.015 per year	[30]
$d_{i}^{US}$	Rate at which an individual in age class *i *dies	0, 0, 0, 0, 0, 0.0167, 0.0750 per year	[30]
$\tau_{i,end}^{US}$	Rate at which a susceptible individual in age class *i *becomes exposed due to travel in endemic countries	1.90 × 10^-3^, 8.06 × 10^-4^, 1.42 × 10^-4^, 8.89 × 10^-5^, 5.94 × 10^-5^, 1.76 × 10^-5^, 1.80 × 10^-5 ^per year	[8,13]
$\beta_{ij}^{US}$	Rate at which an infectious person in age class *j *infects a susceptible person in age class *i *(domestic transmission)	See Appendix	See Appendix; [8,13]
$g_{i}^{US}$	Rate at which a susceptible individual in age class *i *becomes vaccinated	Varies according to scenario	N/a
$N_{i}^{US}$	Population size of age class *i*	20, 20, 40, 40, 40, 60, 40 (millions)	[30]

* The superscript 'US' denotes that the parameter values is speific to the US. Lack of superscript indicates that the same value is used for Canada and the US.

Hepatitis A is not endemic in the US, and the US incidence of HA is only modestly higher than that of Canada. However, there is such a greater volume of travel to the US than to HA-endemic countries that it makes sense to make allowance for infection due to travel to the US in the Canadian model. Hence, the Canadian model is identical except there are three possible sources of infection instead of two: domestic transmission ($\beta_{ij}^{C}$), travel to endemic countries ($\tau_{i,end}^{C}$), and travel to the US ($\tau_{i,US}^{C}$). There are no data on what proportion of reported HA cases in Canada are attributable to travel in the US. Hence, we must estimate $\tau_{i,US}^{C}$ from $\tau_{i,end}^{C}$ using assumptions relating travel volume, duration of stay, and relative incidence levels in the US to the same parameters in endemic countries:

$$\tau_{i,US}^{C}=\tau_{i,end}^{C}\frac{Y^{US}-Y^{C}}{Y^{end}-Y^{C}}\frac{\theta_{i}^{US}}{\theta_{i}^{end}}\frac{\omega_{i}^{US}}{\omega_{i}^{end}}$$

where *Y*^*US *^(resp. *Y*^*end*^, *Y*^*C*^) is the incidence in the US (resp. endemic countries, Canada) where $\theta_{i}^{US}$ (resp. $\theta_{i}^{end}$) is the annual volume of travel by individuals in age class *i *to the US (resp. endemic countries), and where $\omega_{i}^{US}$ (resp. $\omega_{i}^{end}$) is the average duration of stay by travelers in the US (resp. endemic countries). These parameters can be obtained from published data [3] or from travel data available on government agency websites [32]. The difference between Canadian incidence and US/endemic incidence is used since that is proportional to the marginal increase in infection risk to Canadian residents traveling in other countries. For instance, if Canadian incidence were 5 per 100,000 per year, and US incidence changed from 10 per 100,000 per year to 15 per 100,00 per year (due to more foodborne outbreaks in that country, for example), then the additional risk of infection per year that Canadian residents assume upon themselves due to travel to the US would double. We note that Equation 1 does not take all possible factors into account. For instance, individual behaviour may vary, since Canadian residents are perhaps more risk-averse when traveling in an endemic country than when traveling in the US. The Canadian parameters $\tau_{i,end}^{C}$ and $\beta_{ij}^{C}$ are estimated using the same method as for the corresponding US parameters $\tau_{i,end}^{US}$ and $\beta_{ij}^{US}$, except that the force of infection due to travel to the US is first subtracted from the total force of infection, and Canadian seroprevalence, case reporting, and travel data are used (see Appendix B). The resulting values for $\tau_{i,US}^{C}$, $\beta_{ij}^{C}$ and $\tau_{i,end}^{C}$ appear in Table 2.

**Table 2:** Definitions of parameters and variables, and parameter values used for the age-structured Canadian model.*

Variable/Parameter	Definition	Values	Reference
$S_{i}^{C}$	Proportion of susceptible individuals in Canada at a given time *t *in age class *i*	N/A (not applicable)	N/A
$E_{i}^{C}$	Proportion of exposed (infected but not yet infectious) individuals in Canada at a given time *t *in age class *I*	N/A	N/A
$I_{i}^{C}$	Proportion of infectious individuals in Canada at a given time *t *in age class *i*	N/A	N/A
$R_{i}^{C}$	Proportion of recovered individuals in Canada at a given time *t *in age class *i*	N/A	N/A
$V_{i}^{C}$	Proportion of vaccinated individuals in Canada at a given time *t *in age class *i*	N/A	N/A
*a*_i_	Rate at which an individual in age class *i *ages (enters age class *i*+1)	0.2, 0.2, 0.1, 0.1, 0.1, 0.05, 0 per year for age classes 1, 2, ... 7	N/A
*b*^C^	Birth rate	0.015 per year	[43]
$d_{i}^{C}$	Rate at which an individual in age class *i *dies	0, 0, 0, 0, 0, 0.0167, 0.0750 per year	[30]
$\tau_{i,end}^{C}$	Rate at which a susceptible individual in age class *i *becomes exposed due to travel in endemic countries	2.69 × 10^-3^, 1.90 × 10^-3^, 4.04 × 10^-4^, 3.19 × 10^-4^, 2.81 × 10^-4^, 1.14 × 10^-4^, 4.02 × 10^-5 ^per year	[15,43,44,45]
$\tau_{i,US}^{C}$	Rate at which a susceptible individual in age class *i *becomes exposed due to travel in the United States	9.56 × 10^-4^, 6.59 × 10^-4^, 7.89 × 10^-5^, 4.25 × 10^-5^, 4.34 × 10^-5^, 1.77 × 10^-5^, 6.43 × 10^-6 ^per year	[15,43,44,45]
$\beta_{ij}^{C}$	Rate at which an infectious person in age class *j *infects a susceptible person in age class *i *(domestic transmission)	See Appendix	[15,43,44,45]
$g_{i}^{C}$	Rate at which a susceptible individual in age class *i *becomes vaccinated	Varies according to scenario	N/A
$N_{i}^{C}$	Population size of age class *i*	2, 2, 4, 4, 4, 6, 4 (millions)	[43]

* The superscript 'C' denotes that the parameter values is speific to Canada. Lack of superscript indicates that the same value is used for Canada and the US.

The age-structured model for Canada has identical structure to the US model except for the additional term representing infection attributable to travel in the US (see Appendix A). We wish to make the force of infection attributable to travel to the US a function of the number of infectious individuals at any given time in the US, in order to study the effects of differing vaccine coverage in the US and differing travel volume to the US. Hence, instead of $\tau_{i,US}^{C}S_{i}^{C}$, the term in the age-structured Canadian model takes the form $T_{i,US}^{C}S_{i}^{C}$, where

$$T_{i,US}^{C}=\tau_{i,US}^{C}\frac{\sum_{j=1}^{7}I_{j}^{US}}{\sum_{j=1}^{7}{\bar{I}}_{j}^{US,1980-94}}$$

The function $T_{i,US}^{C}$ is the time-varying force of infection attributable to travel to the US and is a function of the model variable $I_{j}^{US}$, the number of infectious individuals of age class *i *at a given time in the US. The quantity ${\bar{I}}_{j}^{US,1980-94}$ is the average number of infectious individuals at any given time in the US in age class *j *in the pre-vaccine era (1980–1994) as predicted by the age-structured model, and $\tau_{i,US}^{C}$ is the force of infection attributable to travel in the US during the pre-vaccine period 1980–2004. The function $T_{i,US}^{C}$ therefore couples the two countries and reflects our assumption that when the number of infected individuals in the US increases (resp. decreases), the number of Canadian individuals becoming infected due to travel in the US also increases (resp. decreases).

The demographic and epidemiological parameter values for Canada are listed in Table 2. Parameter values relating to disease progression in infected individuals are the same as those for the US and so are not listed. Some parameters (such as the birth rate) are very similar in the two countries. The number of residents of the US who become infected while traveling in Canada is likely very small due to the relative population sizes of the two countries, and so a similar term was not introduced in the US equations. However, we note that this assumption could become invalid under certain situations. For instance, if US vaccination coverage is high and Canadian vaccination coverage is low, then travel to Canada could, in principle, be a risk factor for US residents (particularly those living close to the border). However, Figure 1 suggests that this is unlikely in practice, as Canadian incidence has remained below US incidence before and after the vaccine was licensed in both countries in the mid 1990s.

## Results

Here we describe the predicted incidence of hepatitis A in Canada under various vaccination scenarios in the US, and for various volumes of travel to the US. The adjusted incidence values reported here are the predicted incidence of reported cases adjusted for subclinical infection and under-reporting. Hence, the adjusted incidence represents the true incidence of all HA infections. If reported incidence were plotted instead, the qualitative results would be the same and the quantitative results would be similar except for a scaling due to the adjustment for under-reporting and subclinical infection.

**Figure 3:**
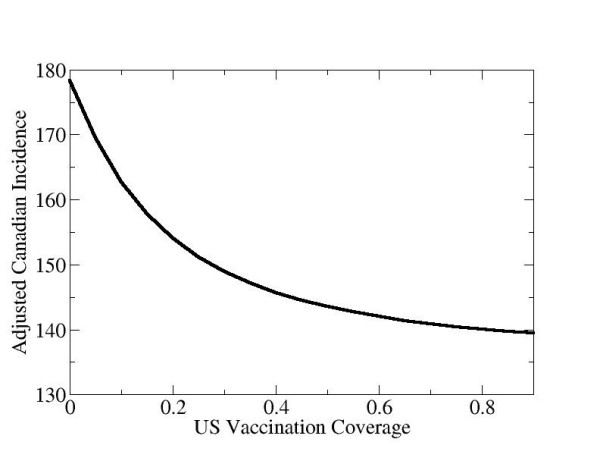
Adjusted incidence in Canada versus US vaccination coverage (in the 0–4 age class), at the equilibrium state of the model dynamics. The adjusted incidence is the reported incidence adjusted for asymptomatic infection and under-reporting.

Figure 3 shows the adjusted incidence in Canada at the equilibrium state of the dynamic model as a function of vaccination coverage in the 0–4 age class in the US. As the vaccine coverage in the US increases, the adjusted HA incidence in Canada decreases significantly. For instance, universal vaccination in the US at 70% coverage in the 0–4 age class causes a 21% decline in the adjusted Canadian incidence, across all age classes. Hence, this allows us to infer that approximately 21% of Canadian incidence was attributable to travel in the United States, in the years for which the model was parameterized (1980 to 1994).

**Figure 4:**
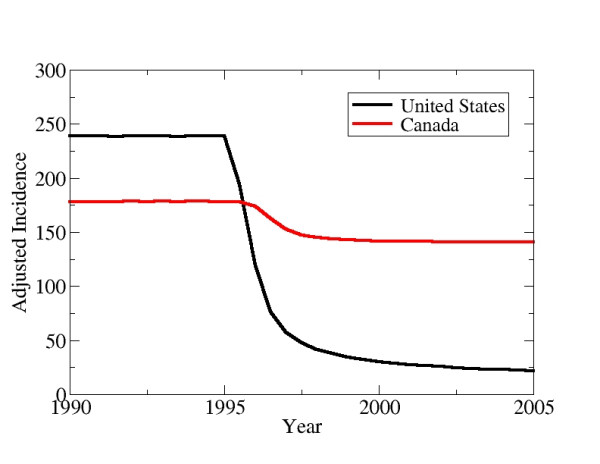
The effect of implementing universal vaccination in the United States on the incidence in Canada. Universal vaccination is implemented in 1995 in the United States by vaccinating 70% of individuals in the 0–4 age class. The adjusted incidence is the reported incidence adjusted for asymptomatic infection and under-reporting.

**Figure 5:**
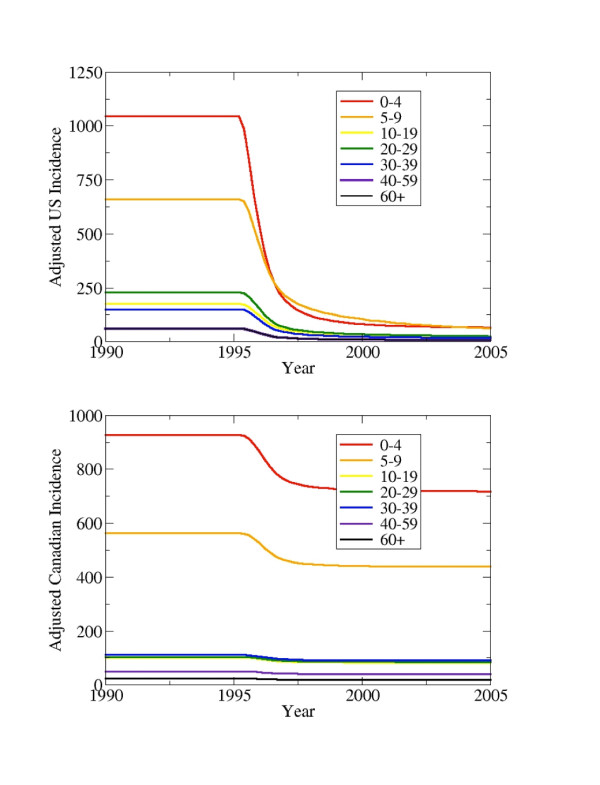
Adjusted incidence by age classes in the US (top) and Canada (bottom) upon initiation of a universal vaccination programme in the US at 70% coverage in all age classes in 1995.

Implementing a universal vaccination programme in the US soon shows its effects in Canadian incidence. Figure 4 shows the adjusted incidence in the US and Canada when, initially, there is no vaccination in either country, but in 1995, a strategy of vaccinating 70% of children in the US in the 0–4 age class begins. Within a few years of the start of the US vaccination programme, adjusted incidence has also declined in Canada significantly. The choice of 1995 as the year that vaccination begins is motivated by the fact that 1994 was the last year before HA vaccine became widely available in the US and Canada. We also note that the model was parameterized using data from 1980 (the earliest year of availability for certain data) to 1994 inclusive. Figure 5 shows the adjusted US and Canadian incidence, stratified by age class, before and after implementing universal vaccination in 1995 at 70% coverage in the 0–4 age class in the US. In the US, incidence declines rapidly not only in the 0–4 age class, but also in the other unvaccinated age classes due to the indirect protective effects of herd immunity. Likewise, incidence declines in all age classes in Canada upon initiation of universal vaccination in the US.

**Figure 6:**
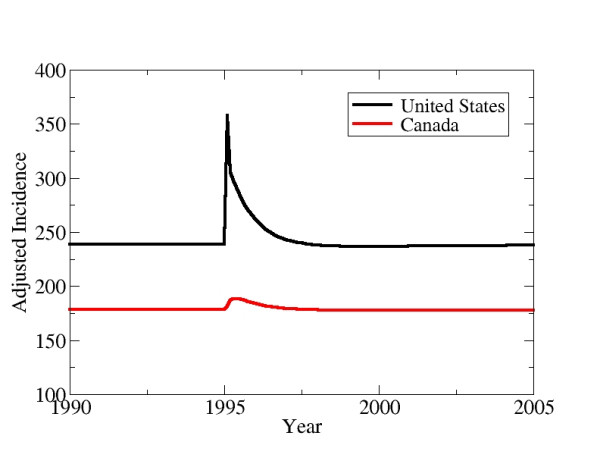
Adjusted incidence in the US and Canada after a sudden increase in US incidence. In 1995, the number of infected individuals in the United States is instantaneously increased by 50%.

Similarly, the effect of an instantaneous 50% increase in the US adjusted incidence in 1995 is soon reflected in a 10% increase in Canadian incidence (Figure 6). Although this scenario of such a rapid increase in incidence is only hypothetical, the example serves to illustrate how closely coupled the countries are. The time difference between the US peak and the Canadian peak in Figure 5 is about 14 days. As noted already, the observed time delay between outbreaks of hepatitis A in gay men in New York with outbreaks in gay men in Montreal is 1–2 months (Vladimir Gilca, INSPQ, pers. comm.). Other scenarios where the effects of fluctuating US incidence on Canadian incidence are studied, such as sinusoidal variation in the US, give rise to lags between US and Canadian incidence peaks of approximately 2 months.

**Figure 7:**
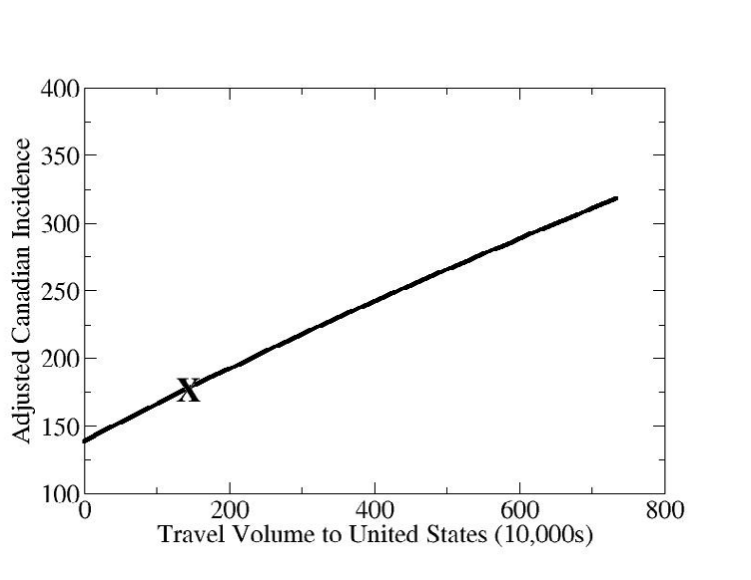
Adjusted incidence in Canada at the equilibrium state of the model dynamics, as a function of the total annual volume of Canadian travel (number of trips per year) to the United States. The "X" denotes the actual volume of travel to the US in 1995.

As the annual volume of travel by Canadian residents to the US increases, the adjusted incidence in Canada also increases in almost direct proportion (Figure 7). The adjusted incidence in Canada when there is no travel to the US is 22% less than the adjusted incidence in Canada at the actual volume of travel in 1995 (indicated in Figure 7). Hence, approximately 22% of infected individuals in Canada in 1995 may have acquired the disease through travel to the US, insofar as Equation 1 is correct.

**Figure 8:**
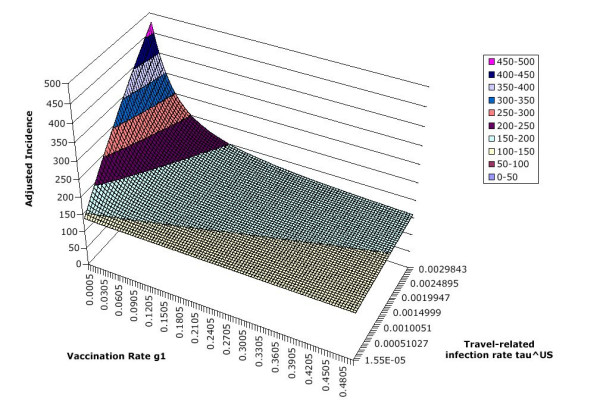
Surface plot of adjusted incidence in Canada, versus vaccination rate $g_{1}^{US}$ and travel-related transmission rate $\tau_{i}^{US}$. Values in legend are adjusted incidence per 100,000 per year.

Figure 8 is a surface plot of adjusted incidence in Canada versus the US vaccination rate ($g_{1}^{US}$) and the force of infection due to travel to the US ($\tau_{i}^{US}$). The travel axis ($\tau_{i}^{US}$) is the weighted average of the $\tau_{i}^{US}$ for all the age classes, which are scaled uniformly to get these results. The plot indicates that both a reduction in travel to the US and an increase in vaccination levels in the US cause a decrease in HA incidence in Canada. If both $\tau_{i}^{US}$ and $g_{1}^{US}$ are large, then small reductions in $\tau_{i}^{US}$ or small increases in $g_{1}^{US}$ have a significant impact on incidence. However, if $\tau_{i}^{US}$ is already small or $g_{1}^{US}$ is already large, reductions in $\tau_{i}^{US}$ and increases in $g_{1}^{US}$ result in much smaller reductions in incidence. Given $\tau_{i}^{US}$ and $g_{1}^{US}$ at some point in time, this plot also indicates the path of steepest descent, i.e., the combination of parameters $\tau_{i}^{US}$ and $g_{1}^{US}$ such that the incidence can be reduced most quickly.

## Discussion

Although HA incidence is much lower in the US than in HA-endemic countries, it is still somewhat higher than in Canada. This, coupled with the enormous annual volume of Canadian travel to the US compared to endemic countries, means that the US could be a more significant source of travel-related infection (particularly for hepatitis A) than previously recognized. Indeed, the results in the previous section illustrate the potential impact of hepatitis A transmission and vaccination in the US on HA prevalence in Canada. Simulation results based on the assumptions in Equation 1 show that a significant proportion (22%) of HA incidence in the mid 1990's, before vaccination was introduced, may have been attributable to travel in the US. Hence, some of the declines in HA incidence observed after 1994 in Canada (Figure 2) may partly be due to the start of universal vaccination in the higher-incidence regions of the US in the mid-1990s.

We speculate that detecting travel to the US as a risk factor is difficult because (1) the incidence is only moderately higher in the US than in Canada (hence the risk to an individual is only modestly increased when traveling to the US), and (2) due to high travel volume, travel to the US becomes a commonplace and under-reported event in the lives of many Canadians. However, this modelling study suggests that future epidemiological studies of risk factors for HA infection should include travel to the US as a variable in risk factor analysis.

There are several limitations to the methodologies used in this paper. Firstly, these results assume the contribution due to travel in the US is similar to the contribution due to travel to endemic countries, adjusted for the total passenger-days in those respective destinations as well as the difference in incidence between Canada and the respective destination (Equation 1). Moreover, here we have neglected cohort effects for the sake of simplicity [33]. In reality, the age-structured seroprevalence profile for hepatitis A exhibits a cohort effect, whereby the seroprevalence in older age classes is higher than can be explained by the current force of infection (thus implying that the force of infection was higher in the past). The existence of a cohort effect influences how the disease can be modeled. In particular, if the cohort effect is neglected and it is assumed that transmission rates have always been constant, then the dynamic model will overpredict the average population incidence both before and after vaccination, and will also overpredict the percentage reduction in incidence due to vaccination [33]. Finally, there is social heterogeneity within countries in risk factors and transmission patterns for hepatitis A that may be important for modelling certain aspects of disease transmission. Geographical heterogeneity in travel destinations of Canadian traveling to the US may also be important.

The actual HA incidence in the US and Canada appeared to oscillate on a non-seasonal seven-year cycle before the vaccine era (Figure 1). However, our model solutions do not oscillate. Non-seasonal oscillations in models are often associated with endemic diseases, and the period of oscillation (time between peaks) can even be predicted successfully from models [31]. In the case of these models, setting the travel transmission rates to zero for both the US and Canada caused the infection to die out, suggesting that hepatitis A was not endemic in these two countries for the period 1980–1994, the time for which the model was parameterized. The fact that HA incidence oscillated during this time suggests that some third endemic region with a natural period of seven years was driving both the US and Canada. This endemic region may be constituted by Mexico, the Caribbean, and Central American countries, with which both the US and Canada have strong travel links. Possible extensions of the present work include a multi-region model where one region is endemic and drives outbreak patterns in other regions. This would allow the model to capture, for instance, US residents who return from endemic countries and travel to Canada shortly thereafter. However, the data requirements for such a model would be significantly greater.

There have been at least eight hepatitis A transmission models in recent years that have assessed hepatitis A transmission and/or vaccination in various populations and have included herd immunity effects [3,14,15,33-37]. Like the present model, these models have mostly been deterministic, age-structured compartmental models. Some have structured the population along social [34] or, like the present study, geographic lines [14,36]. Those models predicting or estimating the effects of universal vaccination report declines in incidence due to universal vaccination that are similar in magnitude to those found by the present model [14,15,36,37]. Several of the models also included a cost-effectiveness or cost-utility analysis [35,37]. Van Effelterre and colleagues included transmission across regions in the United States in a preliminary way, as part of the sensitivity analysis of their model of US hepatitis A transmission and vaccination [36]. They found that the benefits of universal vaccination across the entire US, compared to the benefits of region-specific strategies according to regional HAV incidence, were less important with transmission among regions than without. However, there were still benefits in terms of the number of cases averted by universal vaccination across the entire US with transmission among regions. They did not incorporate travel data into their model. To our knowledge, the present model is the first to incorporate transmission of hepatitis A between countries due to international travel by residents.

The worldwide SARS coronavirus outbreaks exemplified how a public health problem in one population can quickly become a problem in others, due to strong travel connections between countries. The example of hepatitis A transmission in Canada and the US represents the (significantly less spectacular) flipside to that of SARS: the decline of hepatitis A in Canada may partly be attributable to universal vaccination in the US. Other modelling work illustrates how nonvaccinators in a population can "free-ride" by taking advantage of the herd immunity provided by vaccinators [38-41]. This has been compared to a Prisoner's Dilemma (wherein vaccinators are "cooperators" and nonvaccinators are "defectors") and analyzed using game theory [39-41]. In the same way, entire countries can also "free-ride" by benefiting from vaccination programmes carried out in and funded by other countries. As international air travel continues to increase, vaccination policies and public health policies in one country will become increasingly important to other countries. In the future, multi-country or multi-regional models may come into more common usage.

## Conclusion

This study illustrates that changes in hepatitis A vaccination or incidence in the US, or changes in the volume of travel by Canadians to the US, may all have significant and rapidly-realized impacts on the prevalence of hepatitis A in Canada. The possibility of such a connection is also supported by other evidence, such as the positive correlation in hepatitis A incidence in the US and Canada from 1980 to 1994 (Figure 1). Hence, declines in reported incidence since the mid-1990s observed in Canada may be partially attributable to vaccination in the US. Future epidemiological studies of risk factors for HA infection should include travel to the US as a variable in risk factor analysis. Should travel to the US be found as a significant risk factor, then it should be included as such in vaccine recommendations.

## Abbreviations

HA: hepatitis A

US: United States

SEIRV: Susceptible-exposed-infectious-recovered-vaccinated

## Competing interests

The author(s) declare that they have no competing interests.

## Authors' Contributions

All authors contributed to the modelling, parameterization, analysis and/or the writing of the manuscript. RA programmed and simulated the dynamic model and wrote early drafts of the manuscript. AW conceived of the method of parameterization for transmission rates described in Appendix B and wrote early drafts of the manuscript. CTB conceived the study, contributed background material, and finalized the manuscript. All authors read and approved the final manuscript. This study is based on RA's MSc Thesis in Mathematics, University of Guelph, 2006.

## Appendix A: Model Equations

The model equations for the US are:

$$\begin{array}{l} \frac{dS_{i}^{US}}{dt}=-S_{i}^{US}\sum_{j=1}^{7}\beta_{ij}^{US}\frac{I_{j}^{US}}{N_{j}}-\tau_{i,end}^{US}S_{i}^{US}-g_{i}S_{i}^{US}+fV_{i}^{US}-d_{i}S_{i}^{US}+a_{i-1}S_{i-1}^{US}-a_{i}S_{i}^{US}\\ \frac{dE_{i}^{US}}{dt}=S_{i}^{US}\sum_{j=1}^{7}\beta_{ij}^{US}\frac{I_{j}^{US}}{N_{j}}+\tau_{i,end}^{US}S_{i}^{US}-\delta E_{i}^{US}-d_{i}E_{i}^{US}+a_{i-1}E_{i-1}^{US}-a_{i}E_{i}^{US}\\ \frac{dI_{i}^{US}}{dt}=-\gamma_{i}I_{i}^{US}+\delta E_{i}^{US}-d_{i}I_{i}^{US}+a_{i-1}I_{i-1}^{US}-a_{i}I_{i}^{US}\\ \frac{dR_{i}^{US}}{dt}=\gamma_{i}I_{i}^{US}-d_{i}R_{i}^{US}+a_{i-1}R_{i-1}^{US}-a_{i}R_{i-1}^{US}\\ \frac{dV_{i}^{US}}{dt}=g_{i}S_{i}^{US}-fV_{i}^{US}-d_{i}V_{i}^{US}+a_{i-1}V_{i-1}^{US}-a_{i}V_{i}^{US} \end{array}$$

The definitions of the variables and parameters are given in Table 1. Note that *a*_0_*S*_0 _= *bN *to represent recruitment into the youngest age class through birth, and *a*_7 _= 0. The simulations took as initial conditions *S*_0 _= *E*_0 _= *R*_0 _= *V*_0 _= 0 and *I*_0_>0, however, equilibrium solutions are analyzed throughout the results section and hence the initial values are not relevant to the analysis.

The Canadian equations are identical except for the Susceptible and Exposed compartmental equations:

$$\begin{array}{l} \frac{dS_{i}^{C}}{dt}=-S_{i}^{C}\sum_{j=1}^{7}\beta_{ij}^{C}\frac{I_{j}^{C}}{N_{j}^{C}}-\tau_{i,end}^{C}S_{i}^{C}-T_{i,US}^{C}(I_{j}^{US})S_{i}^{C}-g_{i}^{C}S_{i}^{C}+fV_{i}^{C}-d_{i}^{C}S_{i}^{C}+a_{i-1}S_{i-1}^{C}-a_{i}S_{i}^{C}\\ \frac{dE_{i}^{C}}{dt}=S_{i}^{C}\sum_{j=1}^{7}\beta_{ij}^{C}\frac{I_{j}^{C}}{N_{j}^{C}}+\tau_{i,end}^{C}S_{i}^{C}+T_{i,US}^{C}(I_{j}^{US})S_{i}^{C}-\delta E_{i}^{C}-d_{i}^{C}E_{i}^{C}+a_{i-1}E_{i-1}^{C}-a_{i}E_{i-1}^{C} \end{array}$$

The model was simulated in Matlab, and the fourth-order Runge-Kutta method was used to numerically integrate the equations.

## Appendix B: Model Parameterization

The model is parameterized using incidence and demographic data from 1980 to 1994, since seroprevalence data is readily available for years after 1980, and since vaccination introduced in 1995 altered outbreak patterns and hence transmission probabilities.

### US Demographic Parameters

The size of each age class from the 1994 US Census data [30] is approximately *N*_1 _= *N*_2 _= 20,000,000, *N*_3 _= *N*_4 _= *N*_5 _= *N*_7 _= 40,000,000, *N*_6 _= 60,000,000. The number of births in the US in 1994 was approximately 4,000,000 [42].

The ageing parameter, *a*_i_, is simply the inverse of the time spent in each age class, hence *a*_1 _= *a*_2 _= 1/5 = 0.2 year^-1^, *a*_3 _= *a*_4 _= *a*_5 _= 1/10 = 0.1 year^-1^, *a*_6 _= 1/20 = 0.05 year^-1^, *a*_7 _= 0 year^-1^.

The death rates are obtained by requiring the size of each age class to remain constant over time (by balancing the inflow and outflow for each age class). This is expressed by the equations

$$\begin{array}{l} b^{US}N^{US}=a_{1}N_{1}^{US}+d_{1}^{US}N_{1}^{US}\\ \begin{array}{cc} a_{i}N_{i}^{US}=a_{i+1}N_{i+1}^{US}+d_{i+1}^{US}N_{i+1}^{US} & i=1...6 \end{array} \end{array}$$

Solving these equations using the above values for *b*^*US*^, $N_{i}^{US}$, and *a*_i _yields $d_{1}^{US}=d_{2}^{US}=d_{3}^{US}=d_{4}^{US}=d_{5}^{US}=0,d_{6}^{US}=1/60{\text{ year}}^{-1},d_{7}^{US}=3/40{\text{ year}}^{-1}$.

We note that, in principle, it would be possible to include demographic parameters that change over time according to real-world patterns. For instance, the changing sizes of age classes might be incorporated. However, because the primary purpose of the model is to illustrate the effects of travel coupling between the US and Canada rather than to exactly predict future incidence, the introduction of extraneous processes corresponding to the additional parameters may make the model output more difficult to interpret.

### US Clinical Parameters

Clinical and epidemiological literature on Hepatitis A was used to estimate the durations of the latent and infectious periods. The mean duration of the latent period, 1/*δ*, is approximately 2 weeks [27,28]. The mean duration of the infectious period for the different age groups is 1/*γ*_1 _= 3.5 weeks, 1/*γ*_2 _= 3.0 weeks, 1/*γ*_3 _= 1/*γ*_4 _= 1/*γ*_5 _= 1/*γ*_6 _= 1/*γ*_7 _= 2.5 weeks [27,28,36]. The longer durations in younger age classes reflect the fact that virus is shed for longer in children than adults.

### US Transmission Rate Attributable to Travel to Endemic Countries

Let $\lambda_{i}^{US}$ be the total force of infection (the probability per year that a susceptible person in age class *i *becomes infected, or approximately, the number of infected individuals in a given year in age class *i *divided by the susceptibles in that age class at the start of that year). The force of infection consists of contributions from travel to endemic countries and from infection within the US, hence:

$$\lambda_{i}^{US}=\tau_{i}^{US}+\sum_{j=1}^{7}\beta_{ij}^{US}\frac{I_{j}^{US}}{N_{j}^{US}}$$

Also we note that $\tau_{i}^{US}=\kappa_{i}^{US}\lambda_{i}^{US}$, where $\kappa_{i}^{US}$ is the proportion of reported HA cases in the US whose infection can be attributed to travel to endemic countries. Thus $\lambda_{i}^{US}=\tau_{i}^{US}/\kappa_{i}^{US}$. Substituting this into Equation B2, it follows that

$$\sum_{j=1}^{7}\beta_{ij}^{US}\frac{I_{j}^{US}}{N_{j}^{US}}=\frac{\tau_{i}^{US}}{\kappa_{i}^{US}}-\tau_{i}^{US}$$

This is substituted into Equation A1 at equilibrium with no vaccination (i.e. *g*_i _= 0, *f *= 0) to obtain the system of linear equations

$$\begin{array}{l} -\frac{\tau_{i}^{US}}{\kappa_{i}^{US}}S_{i}^{US}-d_{i}^{US}S_{i}^{US}+a_{i-1}S_{i-1}^{US}-a_{i}S_{i-1}^{US}=0\\ \frac{\tau_{i}^{US}}{\kappa_{i}^{US}}S_{i}^{US}-\delta E_{i}^{US}-d_{i}^{US}E_{i}^{US}+a_{i-1}E_{i-1}^{US}-a_{i}E_{i}^{US}=0\\ -\gamma_{i}I_{i}^{US}+\delta E_{i}^{US}-d_{i}^{US}I_{i}^{US}+a_{i-1}I_{i-1}^{US}-a_{i}I_{i}^{US}=0\\ \gamma_{i}I_{i}^{US}-d_{i}^{US}R_{i}^{US}+a_{i-1}R_{i-1}^{US}-a_{i}R_{i}^{US}=0 \end{array}$$

To solve these equations for $\tau_{i}^{US}$, it is necessary to know $\kappa_{i}^{US}$ and $I_{i}^{US}$ (*d*_*i*_, *a*_*i*_, *δ *and *γ *_*i *_are already known).

In 1995, 4.8% of infected HA cases in the US acquired the disease through international travel [13]. Here it is assumed that, when the disease was acquired through international travel, it was acquired by traveling to endemic countries. To obtain age-specific values of the proportion of cases attributable to travel in endemic countries for 1995 (the $\kappa_{i}^{US}$ values to be used in the model), the available age-specific number of cases for 2001 [13] were adjusted for the population sizes of age classes [30] and the resulting incidence values by age for 2001 were multiplied by the ratio of the overall proportion of cases attributable to travel in 1995 compared to 2001, yielding $\kappa_{1}^{US}=\kappa_{2}^{US}=0.1328,\kappa_{3}^{US}=0.0731,\kappa_{4}^{US}=\kappa_{5}^{US}=0.0333,\kappa_{6}^{US}=\kappa_{7}^{US}=0.0254$.

To estimate the number infectious at any given time during the year, $I_{i}^{US}$, we adjust the reported incidence per year per 100,000 in age class *i*, $y_{i}^{US}$, for subclinical infection and under-reporting, yielding the adjusted incidence $Y_{i}^{US}$. This is then modified using the typical duration of infectiousness to obtain $I_{i}^{US}$. The reported incidence per year per 100,000 for the years 1980–1999 is, by age class, $y_{1}^{US}=8.2,y_{2}^{US}=18.7,y_{3}^{US}=12.2,y_{4}^{US}=18.5,y_{5}^{US}=12.2,y_{6}^{US}=4.7,y_{7}^{US}=4.7$[8]. The incidence in each age class is then divided by the probability of jaundice by age class *P*_*i*_: *P*_1 _= 0.11, *P*_2 _= 0.34, *P*_3 _= 0.70, *P*_4 _= *P*_5 _= *P*_6 _= *P*_7 _= 0.81 [15] and multiplied by the under-reporting factor of 10.4 [8] to obtain the adjusted incidence values $Y_{i}^{US}$ ($Y_{i}^{US}$ = 10.4 × observed incidence/*P*_i_). Finally, we must modify $Y_{i}^{US}$ once again since it represents the number of cases per 100,000 per year, not the total number of cases at a given time during the year. For this, following formula is used, $I_{i}^{US}=Y_{i}^{US}N_{i}^{US}/100,000\gamma_{i}$. In other words, the number of infectious individuals at any given time during the year is the annual incidence adjusted for the size of the age group divided by the average number of intervals of infectiousness that can occur within a year. Therefore $I_{1}^{US}=10,456,I_{2}^{US}=6,590,I_{3}^{US}=3,491,I_{4}^{US}=4,560,I_{5}^{US}=3,016,I_{6}^{US}=1,750,I_{7}^{US}=1,167$

Thus the system of linear equations (System B4) can be solved to obtain the values for US transmission rate attributable to travel to endemic countries (see Table 1 for values).

### Domestic US Transmission Rate

Next, $\beta_{ij}^{US}$ is found from Equation B3. Since there are more unknowns (7 × 7 = 49) than knowns (7), we make additional assumptions about the $\beta_{ij}^{US}$ values: $\begin{array}{l} \chi_{1}^{US}=\beta_{11}^{US},\chi_{2}^{US}=\beta_{12}^{US}=\beta_{22}^{US}=\beta_{21}^{US},\\ \chi_{3}^{US}=\beta_{13}^{US}=\beta_{23}^{US}=\beta_{33}^{US}=\beta_{32}^{US}=\beta_{31}^{US},\\ \chi_{4}^{US}=\beta_{14}^{US}=\beta_{24}^{US}=\beta_{34}^{US}=\beta_{44}^{US}=\beta_{43}^{US}=\beta_{42}^{US}=\beta_{41}^{US} \end{array}$, etc. Similar assumptions have been made for other diseases modeled with age-structured compartmental models [31]. Expressed as a matrix, this becomes a "Who Acquires Infection from Whom" matrix. The values obtained by solving the linear system of equations (B3) are found in Table 3.

**Table 3:** Estimated domestic transmission rates in the US.

Parameter	Value
$\chi_{1}^{US}$	9.18 year^-1^
$\chi_{2}^{US}$	5.54 year^-1^
$\chi_{3}^{US}$	1.48 year^-1^
$\chi_{4}^{US}$	2.31 year^-1^
$\chi_{5}^{US}$	1.50 year^-1^
$\chi_{6}^{US}$	0.57 year^-1^
$\chi_{7}^{US}$	0.58 year^-1^

### US Vaccination Parameters

The rate of waning vaccine-derived immunity was obtained by fitting an exponential curve to the estimated proportion of vaccinated individuals retaining immunity after 10, 20, 30, 50 and 70 years (95%, 90%, 81%, 74%, and 68% respectively), obtained using the Delphi method [24], yielding f = 0.005795 year^-1^.

In our simulations, only individuals in the first age class were vaccinated, hence $g_{2}^{US}=g_{3}^{US}=\ldots =g_{7}^{US}=0$. To determine $g_{1}^{US}$ such that 70% of individuals are vaccinated at a given age each year in the 0–4 age class, we set *f *= 0, $\tau_{i}^{US}=0,E_{i}^{US}(0)=I_{i}^{US}(0)=0$, applied the condition

$$\sum_{i=2}^{7}V_{i}^{US}=0.70\sum_{i=2}^{7}N_{i}^{US}$$

to System A1 and solved it analytically to obtain $g_{1}^{US}$ = 7/15 year^-1^.

### Canadian Demographic Parameters

The average size of the age classes from 1980–1994 in Canada were approximately $N_{1}^{C}=N_{2}^{C}=2,000,000,N_{3}^{C}=N_{4}^{C}=N_{5}^{C}=N_{7}^{C}=4,000,000,N_{6}^{C}=6,000,000$[43]. The average number of births per year during this time was approximately 400,000 [43]. The death rates, ageing parameters, and duration of the latent infectious periods are the same as in the US model.

### Canadian Transmission Rate Attributable to Travel to Endemic Countries

The Canadian rate of infection due to travel to endemic countries ($\tau_{i,end}^{C}$) was computed using the same method as for the US, except that there is an additional contribution to the travel transmission rate, $\tau_{i,US}^{C}$, from travel to the US. Hence, the total force of infection in Canada is

$$\lambda_{i}^{C}=\tau_{i,end}^{C}+\tau_{i,US}^{C}+\sum_{j=1}^{7}\beta_{ij}^{C}\frac{I_{j}^{C}}{N_{j}^{C}}$$

Again using the fact that $\tau_{i,end}^{C}=\kappa_{i}^{C}\lambda_{i}^{C}$, we have that

$$\sum_{j=1}^{7}\beta_{ij}^{C}\frac{I_{j}^{C}}{N_{j}^{C}}=\frac{\tau_{i,end}^{C}}{\kappa_{i}^{C}}-\tau_{i,end}^{C}-\tau_{i,US}^{C}$$

$\tau_{i,end}^{C}$ is found following the same method as for the US case, but using Canadian incidence and travel data. The values appear in Table 2.

### Canadian Transmission Rate Attributable to Travel to the US

The parameter $\tau_{i,US}^{C}$ is obtained from Equation 1. The volume of travel (annual number of trips) by Canadian residents using any mode of transport in 1995 to the US and endemic countries is available from published data [16]. Estimates of age-specific travel volumes to the US ($\theta_{i}^{US}$) and endemic countries ($\theta_{i}^{end}$) are obtained from these data by assuming travel volume to be distributed across the age classes according to the sizes of the age classes [43], yielding the results in Table 4.

**Table 4:** Travel volume (number of trips per year) to the US and to HA-endemic countries, 1995.

Parameter	Value	Parameter	Value
$\theta_{1}^{US}$	554,000	$\theta_{1}^{end}$	33,000
$\theta_{2}^{US}$	542,000	$\theta_{2}^{end}$	33,000
$\theta_{3}^{US}$	767,000	$\theta_{3}^{end}$	83,000
$\theta_{4}^{US}$	1,553,000	$\theta_{4}^{end}$	246,000
$\theta_{5}^{US}$	2,627,000	$\theta_{5}^{end}$	360,000
$\theta_{6}^{US}$	5,559,000	$\theta_{6}^{end}$	757,000
$\theta_{7}^{US}$	3,061,000	$\theta_{7}^{end}$	404,000

The average duration of travel to the US and to endemic regions are $\omega_{i}^{US}$ = 7.20 nights and $\omega_{i}^{end}$ = 18.30 nights respectively [16]. The incidence per 100,000 per year in the US, Canada and endemic countries, adjusted for under-reporting and subclinical infection, are estimated as *Y*^US ^= 215.56, *Y*^C ^= 156.83, *Y*^End ^= 1250.00 [3,8,15]. All these values are substituted into Equation 2 to obtain the values appearing in Table 2.

### Local Canadian Transmission rate

The parameter $\beta_{ij}^{C}$ is found using the same methods as for the US model, yielding values for $\chi_{i}^{C}$ (see Table 5).

**Table 5:** Estimated domestic transmission rates in Canada.

Parameter	Value
$\chi_{1}^{C}$	5.54 year^-1^
$\chi_{2}^{C}$	3.25 year^-1^
$\chi_{3}^{C}$	0.62 year^-1^
$\chi_{4}^{C}$	0.88 year^-1^
$\chi_{5}^{C}$	1.04 year^-1^
$\chi_{6}^{C}$	0.47 year^-1^
$\chi_{7}^{C}$	0.24 year^-1^

### Canadian Vaccination Parameters

The rate of loss of vaccine derived immunity is the same as for the US model, f = 0.005795 year^-1 ^[24]. The vaccination policy in Canada is currently targeted vaccination toward high-risk groups. A previous study estimates that, to date, about 7% of the Canadian population has been vaccinated under this programme (which also vaccinates travelers to endemic countries) (Bauch et al, unpublished data). Hence, we assume in this paper a 7% coverage rate for each age class. Although the actual coverage rates across age classes under the current targeted policy may be dissimilar, the available data do not allow us to stratify the vaccine coverage rates by age. Hence, we have assumed the same vaccination rate applies to each age class. We note that there are also other heterogeneities in vaccine coverage (e.g., social) that the present model was not designed to address. The values of *g*_i _are obtained from imposing *f *= 0, $\tau_{i}^{C}=\tau_{i}^{US}=0$ as for the US case, as well as the constraints

$$\begin{array}{ll} \sum_{i=1}^{7}V_{i}^{C}=0.07\sum_{i=1}^{7}N_{i}^{C} & \\ g_{i}^{C}=g^{C} & \begin{array}{cc} for & i=1..7 \end{array} \end{array}$$

on System A2, and solving System A2 to yield *g*^C ^= 0.001937 year^-1^.

## Pre-publication history

The pre-publication history for this paper can be accessed here:


